# Prevalence and Correlates of Binge Drinking among Young Adults Using Alcohol: A Cross-Sectional Survey

**DOI:** 10.1155/2014/930795

**Published:** 2014-06-30

**Authors:** Francesco Bartoli, Daniele Carretta, Cristina Crocamo, Alessandro Schivalocchi, Giulia Brambilla, Massimo Clerici, Giuseppe Carrà

**Affiliations:** ^1^Department of Surgery and Translational Medicine, University of Milano Bicocca, Via Cadore 48, 20900 Monza, Italy; ^2^Division of Psychiatry, Faculty of Brain Sciences, University College London, Charles Bell House, 67–73 Riding House Street, London W1W7EJ, UK

## Abstract

*Background*. Although binge drinking prevalence and correlates among young people have been extensively studied in the USA and Northern Europe, less is known for Southern Europe countries with relatively healthier drinking cultures. *Objective*. We aimed at analyzing prevalence and correlates of binge drinking in a representative sample of young adults in Italy. *Methods*. We conducted a cross-sectional survey among alcohol-consuming young adults. We carried out univariate and multivariate analyses to assess associations between recent binge drinking and candidate variables. *Results*. We selected 654 subjects, with 590 (mean age: 20.65 ± 1.90) meeting inclusion criteria. Prevalence for recent binge drinking was 38.0%, significantly higher for females than males. Multivariate analysis showed that high alcohol expectancies, large amount of money available during the weekend, interest for parties and discos, female gender, cannabis use, influence by peers, and electronic cigarettes smoking all were significantly associated with recent binge drinking, whereas living with parents appeared a significant protective factor. *Conclusions*. More than a third of young adults using alcohol are binge drinkers, and, in contrast with findings from Anglo-Saxon countries, females show higher risk as compared with males. These data suggest the increasing importance of primary and secondary prevention programmes for binge drinking.

## 1. Introduction

Binge drinking can be described as heavy alcohol use over a short period of time [1], and it is typically defined by a consumption of four or five drinks in a row among women and men, respectively [2]. This dangerous pattern of alcohol consumption is highly prevalent among young adults and a public health concern in the USA [3] as well as in most of European countries [4]. Data from the 2001 National Household Survey on Drug Abuse on 19–21-year-old US adults highlighted a weekly binge drinking prevalence of 12% and 27% among females and males, respectively [5]. At the same time, relevant research shows that there is an increase of binge drinking among young people also across Europe [6, 7]. A six European countries (Germany, Iceland, Italy, Netherlands, Poland, and Scotland) cross-sectional survey on 16,551 pupils from 114 public schools showed that 27% of the sample had consumed >5 drinks in a row on at least 1 occasion in their life [8]. Pleasure, habit, increasing confidence, anxiety or stress, and social pressures have been reported as the most common reasons for alcohol drinking during adolescence and early adulthood [9]. Furthermore, the impact of binge drinking among young people has been associated with an increased risk of social and clinical consequences in the adulthood, such as illicit drug use, psychiatric morbidity, homelessness, convictions, school exclusion, lack of qualifications, and accidents [10–12]. Indeed, alcohol dependence in young adults is often preceded by higher persisting rates of frequent, intense, or binge drinking [13]. Adolescents and young adults who engage in binge drinking are more likely to report other health risk behaviors [14], such as smoking cigarettes and/or cannabis [15–18]. In a sample with a modal age of 18 years both cigarettes and marijuana use predicted both binge drinking and an extreme level of binge drinking, defined as ≥15 drinks in a row [19]. Also, comorbid mental disorders might play a role in alcohol misuse risk [20].

However, studies on binge drinking characteristics and correlates conducted in Southern Europe are sparse [21–24], though relatively healthier drinking culture might moderate magnitude and consequences of excessive alcohol intake among young people [25]. Furthermore, there is a lack of research exploring prevalence and correlates of binge drinking in natural settings, whereas most of studies were specifically conducted among high school [14, 23] or college/university students [2, 21, 22]. With a view of remedying these limitations, the aim of this cross-sectional study was to explore prevalence and correlates of binge drinking in a representative sample of not abstemious young adults recruited in area of the Milan nightlife scene.

## 2. Methods

### 2.1. Study Design

We conducted a cross-sectional survey on young adults aged between 18 and 24 years, drawn up according to the STrengthening the Reporting of OBservational studies in Epidemiology (STROBE) Statement - Checklist [26]. The Ethics Committee of University of Milano Bicocca approved the study.

### 2.2. Participants and Peer Interviewers

We consecutively recruited not abstemious young adults aged between 18 and 24 years. Those who joined the study received an information sheet and signed a written consent. We included only individuals able to sign the informed consent and excluded people who self-reported consumption of alcohol or drugs. The interviewers were students, peers aged between 18 and 24 years, selected from different Schools of Milano Bicocca University, and receiving 10 hrs training for the research project about data collection procedures, including checking eligibility, providing information on the research project, obtaining consent, and distributing and assisting with questionnaires. Questionnaires were administered through a Smartphone application. Subjects who accepted to participate to the study received a €10.00 mobile phone recharge.

### 2.3. Settings

Recruitment took place in urban locations of Milan nightlife scene, an area of about 1.3 million of inhabitants. We choose areas with a high density of pubs, clubs, discos, or live music events. The recruitment period was between April and July, 2013.

### 2.4. Measures

Recent binge drinking was assessed by a single question exploring if a consumption of four or five drinks in a row among females and males, respectively, occurred at least once, in the last two weeks. In order to analyze potential factors associated with binge drinking, we identified from scientific literature [5, 27–30] relevant candidate correlates, building a specific 10-minute questionnaire including questions on sociodemographic, clinical, individuals, and lifestyle characteristics, as well as substance and alcohol-related behaviors.

*(a) Sociodemographic Characteristics*. We collected information on age, gender, country of birth, living condition, relationship, educational and employment status, and financial availability for each weekend.

*(b) Clinical and Individual Characteristics.* As there is a strong association between substance-related behaviors and mental disorders [31–33], particularly between binge drinking and depression [34], we investigated presence of a depressive disorder with a yes/no single item screening question:* have you felt depressed or sad much of the time in the past year?* We screened for anxiety symptoms in the same manner, asking:* have you felt anxious much of the time in the past year?* Single-item questions have been used for depressive disorders screening in both general and clinical populations [35, 36], as well as for anxiety detection [37]. In order to assess impulsivity, likely associated with the risk of binge drinking among young adults [38, 39], we explored its levels using specific items of Substance Use Risk Profile Scale (SURPS) [40]. The SURPS subscale for impulsivity is a five-item questionnaire developed for use in adolescents, and it has been correlated with alcohol abuse and physiological dependence symptoms [40].

*(c) Lifestyle Characteristics*. First, we explored the interest for joining events and attending recreational settings where it may be easier engaging in binge drinking, such as night parties or discos [27, 41]. Second, due to the high rates of alcohol use in athlete populations [42], we checked the involvement in sport activities. Finally, we explored religiosity that, on the other hand, may represent a potential protective factor [43].

*(d) Substance and Alcohol-Related Behaviors*. We analyzed substance-related behaviors potentially associated with binge drinking [16, 44], collecting information on habits of smoking nicotine cigarettes and/or cannabis during the last 30 days. Furthermore, we checked also for electronic cigarettes (e-cigarettes), a growing phenomenon among young adults [45, 46]. Moreover, we investigated the potential influences of peers on the risk of binge drinking, which may represent important factors associated with binge drinking [28], asking if most of close friends were alcohol drinkers or abstainers. Then, we collected information on the age of onset of alcohol drinking, considering an early onset if it happened before 17 years. Finally, we explored if the subject had high alcohol expectancies for social facilitation [39] through the Alcohol Expectancies Questionnaire for Adolescents, Brief (AEQ-AB) [47]. The AEQ-AB is a seven-item Likert scale exploring alcohol expectancies on global positive changes, changes in social behavior, improved cognitive and motor abilities, sexual enhancement, cognitive and motor impairment, increased arousal, and relaxation and tension reduction [47].

### 2.5. Statistical Analysis

Statistical analyses were performed using Stata version 10.0 SE. We carried out univariate analyses to identify attributes characteristic of people who binge drink. The normality of continuous data was checked with Shapiro-Wilk's test. Student's *t*-test was performed for normally distributed continuous data. If normality assumption was rejected for dependent variable distribution, we used nonparametric Wilcoxon-Mann-Whitney test. Chi-square and Fisher's exact tests were used for categorical variables. We identified covariates significantly associated (*P* < 0.05) with binge drinking for inclusion in subsequent multivariate analyses. Association with binge drinking was shown as odds ratio (OR) with related 95% confidence intervals (CI) and *P* value. We carried out logistic regressions, controlling for age and sex (as well as adjusting for clusters related to specific place of recruitment) and for variables that were significantly related to binge drinking in the univariate analyses.

## 3. Results

### 3.1. Participants

We recruited 654 potentially eligible subjects, with 590 (90.2%) meeting our inclusion criteria. No eligible individual refused to participate in the study. The sample comprised 286 males (48.5% of sample) and 304 females (51.5%). The mean age (±standard deviation) was 20.65 ± 1.90, with no significant gender differences. Most of selected subjects were born in Italy, lived with parents, were single in their undergraduate higher education, and not yet in formal employment. Sociodemographical characteristics of the sample are fully described in Table 1.

For most of the variables, we had no missing data, although for possibly sensitive items of questionnaires response rates ranged from 96.3% (*being in a relationship*) to 99.5% (*religiosity*;* living alone or with parents*;* anxiety*;* depression*;* financial availability for each weekend*).

### 3.2. Univariate Analysis

**Figure 1:**
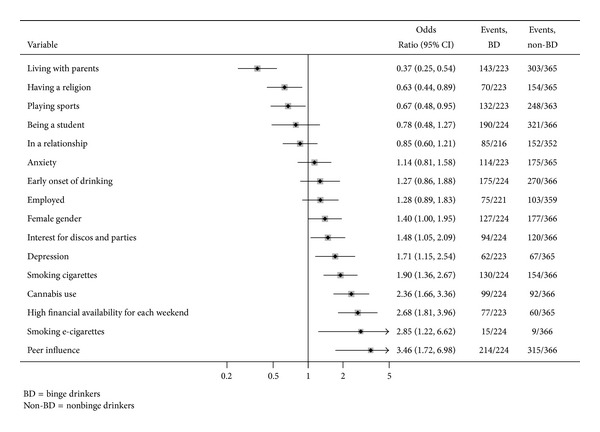
Correlates of binge drinking: univariate analysis of categorical variables.

People with a recent binge drinking were 224 (38.0% of the sample). Rates of a recent binge drinking were significantly higher among females than males (41.8% versus 33.9%; *P* = 0.049). Binge drinkers were significantly older than their nonbinge drinking counterpart (21.1 ± 1.9 versus 20.3 ± 1.9 years; *P* < 0.001). People who recently engaged in binge drinking showed higher scores of both impulsivity (*P* < 0.001) and alcohol expectancies (*P* < 0.001) according to SURPS and AEQ-AB scales, respectively. Furthermore, depressive disorders were more frequent among binge drinkers than in nonbinge drinking individuals (27.8% versus 18.4%; *P* = 0.007), but no statistical differences were found for what concerns anxiety symptoms (51.1% versus 47.9%; *P* = 0.455). All smoking habits appeared significantly related to a recent binge drinking episode: cigarettes, cannabis, and e-cigarettes were regularly used by 58.0%, 44.2%, and 6.7% of binge drinkers, and by 42.1%, 25.1%, and 2.5% of nonbinge drinkers, respectively. Univariate analyses showed that having a high interest for parties and discos (*P* = 0.024), having more than €50 available* per* weekend (*P* < 0.001), and having most of friends drinking alcohol (*P* < 0.001) all were significantly associated with a recent binge drinking episode. On the other hand, young people living with parents (*P* < 0.001), playing sport (*P* = 0.025), or who claimed to be religious (*P* = 0.009) were less likely to have been recently engaged in binge drinking. Univariate analyses for categorical and continuous variables are detailed in Figure 1 and Table 2.

### 3.3. Multivariate Analysis

We performed multivariate analysis, taking into account age, gender, place of recruitment, and all variables significantly associated with binge drinking at univariate analyses. Results are described in Table 3. Positive alcohol expectancies (*P* < 0.001), a high financial availability for each weekend (*P* = 0.041), interest for parties and discos (*P* < 0.006), female gender (*P* < 0.001), cannabis use (*P* = 0.003), influence by peers (*P* < 0.001), and e-cigarettes smoking (*P* = 0.047) all were significant correlates of binge drinking. On the other hand, living with parents appeared significantly protective (*P* = 0.031). Other variables included in the multivariate analysis, such as age (*P* = 0.127), cigarettes smoking (*P* = 0.841), impulsivity (*P* = 0.229), playing sport (*P* = 0.506), religiosity (*P* = 0.333), depressive symptoms (*P* = 0.384), were not significantly associated with the likelihood of a recent episode of binge drinking.

## 4. Discussion

### 4.1. Summary and Interpretation of Findings

This study describes the prevalence and correlates of binge drinking in a large representative sample of Italian alcohol consuming young adults, recruited in the Milan night scene.

Binge drinking prevalence was 38%, significantly higher among females than males, as confirmed by the multivariate analysis. These rates are slightly higher than those reported by previous studies focused exclusively on Italian university students [21, 22]. Furthermore, surprisingly enough, females appeared more likely to have been engaged in a recent episode of binge drinking than males. This finding is different from results of similar studies, for example, [21, 27], in which being male appeared significantly associated with the risk of both recent and lifetime binge drinking. These differences may be explained not only by several conditions, including our settings, recruitment sources, and sociodemographic characteristics, but also by recent changes in drinking habits among young people. Heavy episodic drinking is becoming quite common also in Southern Europe countries, despite their traditional daily but moderate alcohol consumption [25]. Indeed, recent studies report that young women have begun to show drinking patterns similar to those of their male peers, especially regarding heavy episodic drinking [48, 49]. Furthermore, it should be noted that we included in our study only young alcohol consumers, excluding alcohol abstainers. Therefore, both high prevalence and gender difference of binge drinking in our sample may be influenced by the fact that we have analyzed only a subpopulation of young adults at risk for dangerous alcohol-related behaviors. We could hypothesize that, despite the fact that 18–24-year-old females are more often alcohol abstainers than males, according, for example, to evidence on European alcohol consumption patterns [50], consuming-alcohol females have a greater risk of being engaged in binge drinking, at least in our geographical area and settings. However, larger studies should be performed to confirm this quite striking result. Our findings show also that binge drinking is significantly related to financial availability, high expectancies from alcohol, use of cannabis, peer influence on drinking patterns, and interest for discos and parties, as similarly reported in other publications of the last 20 years [6, 27, 29, 51–53]. Moreover, this is one of the first studies analyzing the prevalence of the emerging phenomenon of e-cigarettes use [54] in a sample of young adults [45, 46] and its relationship with alcohol risk behaviors. Our study highlighted that smoking electronic cigarettes is significantly associated with a recent episode of binge drinking. However, this finding should be interpreted with caution, since the prevalence of e-cigarettes users in the whole sample was low, corresponding to 6.7% and 2.5% among binge and nonbinge drinkers, respectively. Further studies could be useful to understand if e-cigarettes could be a marker for proneness or vulnerability to sensation seeking and substance consumption, including alcohol and nicotine, given their widespread use among young nonsmokers [55], which is often not part of a smoking quit plan [56]. Living with parents was the only significant protective factor, confirming results from other studies reporting that living outside parental home and far from parental control is associated with a higher risk of binge drinking [57]. None of remaining variables, depressive and anxiety symptoms, cigarettes smoking, impulsive traits of personality, playing sport, and being religious, was statistically associated with recent binge drinking.

### 4.2. Strengths and Limitations

The main strength of this study is based on representative sampling procedures in natural settings of nightlife scene. Furthermore, any effort to enhance young people participation has been implemented, including peer interviewers, use of smartphone, and financial incentive. This may explain the high response rates. However, also several limitations need to be acknowledged, first the cross-sectional design, which prevents from identifying causal links between binge drinking and related factors. Moreover, in the quick, 10-minute-questionnaire, a number of sensitive correlates (e.g., average amount of alcohol use, exposure to previous traumatic events, family habits with drinking, and consumption of drugs other than nicotine and cannabis) had to be excluded, though the literature has shown relevant correlations with binge drinking [6, 58, 59]. For the same reason, we did not use standardized interviews to detect mental disorders, and the anxiety and depression symptoms were checked only with a single item question, though psychometric properties of this approach are quite satisfactory [35]. Finally, since our survey was based on a single urban area of Northern Italy, the results of our study may not be generalizable to the whole Italian general population of young adults.

### 4.3. Conclusions

More than a third of young adults using alcohol from our sample were recent binge drinkers. This is slightly higher than rates of other studies from similar geographical areas [21, 22], representing a potential serious risk to public health, because of high risk of adverse consequences [14]. There is the need of developing specific interventions for primary and secondary prevention, and public health research should focus on new programs that may reduce rates of binge drinking or, at least, its adverse health outcomes [27, 60]. At the same time, prevention strategies should be linked with changes of general public attitudes often considering binge drinking as an acceptable “rite of passage” of adolescents and young adults, with fewer dangerous health consequences than other illicit substance-related risky behaviors [3].

## Figures and Tables

**Table 1:** Sociodemographic characteristics.

Variable	Cases (*n*)	Prevalence (%)
Gender		
Male	286	48.5
Female	304	51.5
Age		
18-19 yrs	178	30.2
20–22 yrs	300	50.8
23-24 yrs	112	19.0
Place of birth		
Italy	547	92.7
Abroad	43	7.3
Living with parents		
Yes	446	75.9
No	142	24.1
In a relationship		
Yes	237	41.7
No	331	58.3
Educational status		
Nonstudent	79	13.4
High school student	162	27.5
University student	349	59.1
Full- or part-time work		
Yes	178	30.7
No	402	69.3

**Table 2:** Correlates of binge drinking: univariate analysis of continuous variables.

Variable	BD (=224)		Non-BD (=366)		Test	OR (95% CI)	*P*
	Mean	SD	Mean	SD			
Age	21.15	1.87	20.35	1.86	−5.05^a^	1.25 (1.14–1.36)	<0.001
Alcohol expectancies	22.49	3.73	20.79	3.72	−5.33^b^	1.12 (1.08–1.17)	<0.001
Impulsivity	5.55	2.27	4.87	1.96	−3.87^a^	1.17 (1.08–1.26)	<0.001

BD: binge drinkers and non-BD: nonbinge drinkers.

^a^Student's *t*-test: *t*.

^b^Mann-Whitney test: *z*.

**Table 3:** Correlates of binge drinking: multivariate analysis*.

Variable	OR	Robust	*P*
	(95% CI)	Std. Err.	
Age	1.19 (0.95–1.48)	0.133	0.127
Female gender	1.57 (1.41–1.75)	0.087	<0.001
Living with parents	0.57 (0.34–0.95)	0.149	0.031
High financial availability for each weekend	1.33 (1.01–1.74)	0.183	0.041
Depression	1.29 (0.73–2.28)	0.375	0.384
Cannabis use	1.61 (1.18–2.20)	0.256	0.003
Smoking cigarettes	1.04 (0.74–1.46)	0.181	0.841
Smoking e-cigarettes	2.49 (1.01–6.18)	1.154	0.047
Impulsivity	1.08 (0.95–1.22)	0.066	0.229
Positive alcohol expectancies	1.11 (1.09–1.13)	0.009	<0.001
Peer influence	2.40 (1.71–3.37)	0.417	<0.001
Interest for discos and parties	1.53 (1.13–2.06)	0.233	0.006
Playing sports	0.93 (0.75–1.15)	0.103	0.506
Having a religion	0.77 (0.45–1.31)	0.209	0.333

*Adjusted for age, gender, and specific place of recruitment.
